# New targets to alleviate skeletal muscle inflammation: role of microRNAs regulated by adiponectin

**DOI:** 10.1038/srep43437

**Published:** 2017-02-27

**Authors:** Raphaël Boursereau, Michel Abou-Samra, Sophie Lecompte, Laurence Noel, Sonia M. Brichard

**Affiliations:** 1 Endocrinology, Diabetes and Nutrition Unit, Institute of Experimental and Clinical Research and Medical Sector, Catholic University of Louvain, 1200 Brussels, Belgium

## Abstract

Muscle inflammation worsens metabolic disorders as well as devastating myopathies. The hormone adiponectin (ApN) has emerged has a master regulator of inflammation/immunity in several tissues including the skeletal muscle. In this work, we explore whether microRNAs regulated by ApN may represent novel mechanisms for controlling muscle inflammation. By screening arrays, we found miR-711 as a strong candidate for mediating ApN action. Thus, ApN-knockout mice showed decreased muscular expression of miR-711 together with enhanced inflammation/oxidative stress markers, while mice overexpressing ApN showed increased miR-711 levels. Likewise, electrotransfer of the ApN gene in muscle of ApN-knockout mice upregulated miR-711 while reducing inflammation and oxidative stress. Similar data were obtained in murine C2C12 cells or in human primary myotubes treated with ApN. MiR-711 overexpression downregulated several components of the Toll-like receptor-4 (TLR4) pathway, which led to repression of NF-κB activity and downstream pro-inflammatory cytokines. MiR-711 blockade had opposite effects. Moreover, muscle electrotransfer of pre-miR-711 recapitulated *in vivo* the anti-inflammatory effects observed *in vitro*. Thus, miR-711, which is upregulated by ApN represses TLR4 signaling, acting therefore as a major mediator of the anti-inflammatory action of ApN. This novel miRNA and its related target genes may open new therapeutic perspectives for controlling muscle inflammation.

Chronic muscle inflammation may be present as either a low-grade or a severe form. The low-grade form characterizes metabolic disorders. Thus, obesity induces a low-grade and chronic state of inflammation in muscle, which contributes to insulin resistance, muscle being the dominant organ for insulin-stimulated glucose disposal^1^,^2^. On the other hand, a more aggressive form may play a role in the development of myopathy and in disease severity. In dystrophic skeletal muscle (Duchenne Muscular Dystrophy being the most devastating type), part of the accumulating muscle damage is caused by pronounced and ongoing activation of inflammatory cells rather than by direct mechanical injury^3^,^4^. Resetting the immunological balance in skeletal muscle may therefore be crucial for the management of such disorders.

Adiponectin (ApN) is a hormone abundantly secreted by adipocytes under normal conditions. Circulating ApN is decreased in obese individuals and in patients with the metabolic syndrome^5^. Besides its metabolic (mainly insulin-sensitizing and fat-burning) properties, ApN has emerged as a master regulator of inflammation/immunity in a variety of tissues including the skeletal muscle, and it thus able to counteract different aspects of the metabolic syndrome as well as excessive inflammatory responses^6^,^7^. We have recently reported that ApN was sufficiently powerful to offset severe inflammation/oxidative stress and muscle damage in dystrophic muscles of mdx mice (a model of Duchenne Muscular Dystrophy)^8^. Conversely, muscles of mice with complete ApN deficiency (ApN Knock-Out, ApN-KO), which were first described as susceptible to insulin-resistance^9^, also displayed high muscular vulnerability to inflammation/oxidative stress and apoptosis. These abnormalities were further worsened by an acute or a chronic inflammatory challenge (Lipopolysaccharide (LPS) injection or high-fat diet) but were then corrected after local electrotransfer of the ApN gene^10^,^11^. As yet, the mechanisms by which ApN shifts the immune balance of muscle cells toward a less inflammatory phenotype are not fully elucidated.

MicroRNAs (miRNAs) are small noncoding RNAs that control gene expression by inducing target mRNA degradation or blocking translation. Dysregulation of miRNAs has been implicated in the metabolic syndrome or in other inflammatory process^12^,^13^. The miRNAs, which serve as mediators for the anti-inflammatory action of ApN have been scarcely studied. We have previously shown that miR-883b-5p and miR-1934, which were upregulated by ApN, were anti-inflammatory factors in adipose tissue^14^,^15^. Likewise, miR-155, which was also upregulated by ApN, played an important anti-inflammatory role in RAW 264.7 macrophages^16^. So far, whether some miRNAs mediate the potent anti-inflammatory/immunomodulatory action of ApN on muscle is still unestablished.

The aim of the present work was to address this important question. To this end, we first took advantage of ApN-KO mice submitted or not to muscle electrotransfer of the ApN gene to identify miRNAs regulated by ApN *in vivo*. Second, we characterized the functions of these novels miRNAs by *in vitro* and *in vivo* experiments. Finally, we also examined whether these miRNAs were regulated by ApN in human.

## Results

### Muscle electrotransfer of ApN gene prevents LPS-induced inflammation and oxidative stress in ApN-KO mice

**Figure 1:**
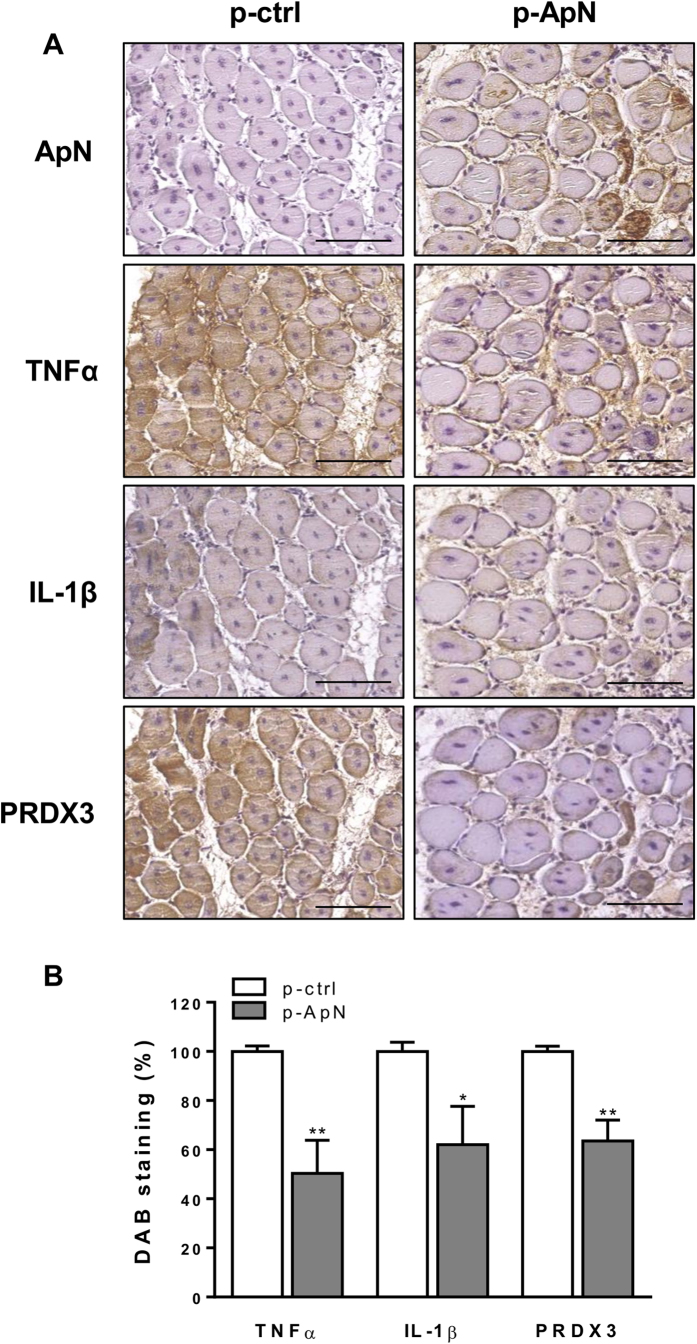
Effects of adiponectin gene electrotransfer on different markers of inflammation and oxidative stress in skeletal muscles of ApN-KO mice. One *tibialis anterior* muscle of ApN-KO mice was injected and electroporated with ApN cDNA-containing plasmid (p-ApN), whereas the contralateral muscle was injected and electroporated with a control plasmid (p-ctrl). Nine days later, mice were challenged by LPS and the *tibialis anterior* muscles were sampled 24 h later. (**A**), Immunochemistry performed with specific antibodies directed against adiponectin (ApN), two pro-inflammatory cytokines (TNFα, IL-1β) and an oxidative stress marker (PRDX3). Scale bar = 100 μm. (**B**), Quantification of DAB staining areas within muscles. Results are means ± SEM for 5 mice (the contralateral muscle being used as control). *P < 0.05, **P < 0.01 for the effect of the ApN gene.

We first confirmed our previous data^10^: local administration of ApN was able to protect ApN-KO mice against LPS-induced muscular damage (Fig. 1). One *tibialis anterior* muscle was injected with a plasmid containing the ApN sequence, while the contralateral one received an empty plasmid; muscles were then electroporated. Nine days later, mice were challenged by intraperitoneal injection of LPS.

As expected, electrotransfer of the ApN gene induced a local expression of ApN in the target muscle of ApN-KO mice (Fig. 1A). Concomitantly, there was no expression of the adipokine in the contralateral muscle and no rise of ApN concentrations in the plasma where the levels remained undetectable (not shown). Muscle electrotransfer of the ApN gene reduced the expression of two inflammatory cytokines (tumor necrosis factor alpha; TNFα and interleukine 1 beta; IL-1β) and of one oxidative stress marker (peroxiredoxin 3; PRDX3), when compared with the contralateral untreated muscle. This reduction averaged 40 to 50% as shown by quantification of 3,3′-Diaminobenzidine (DAB) staining (Fig. 1B). These results clearly confirmed the beneficial local effect of ApN on inflamed muscle^10^,^11^. This effect may operate in a paracrine manner as serial muscle cross-sections revealed that the extension of this anti-inflammatory action was wider than the localized expression of the hormone.

### MiRNA expression profiling in muscle of ApN-KO mice after electrotransfer of ApN gene

**Figure 2:**
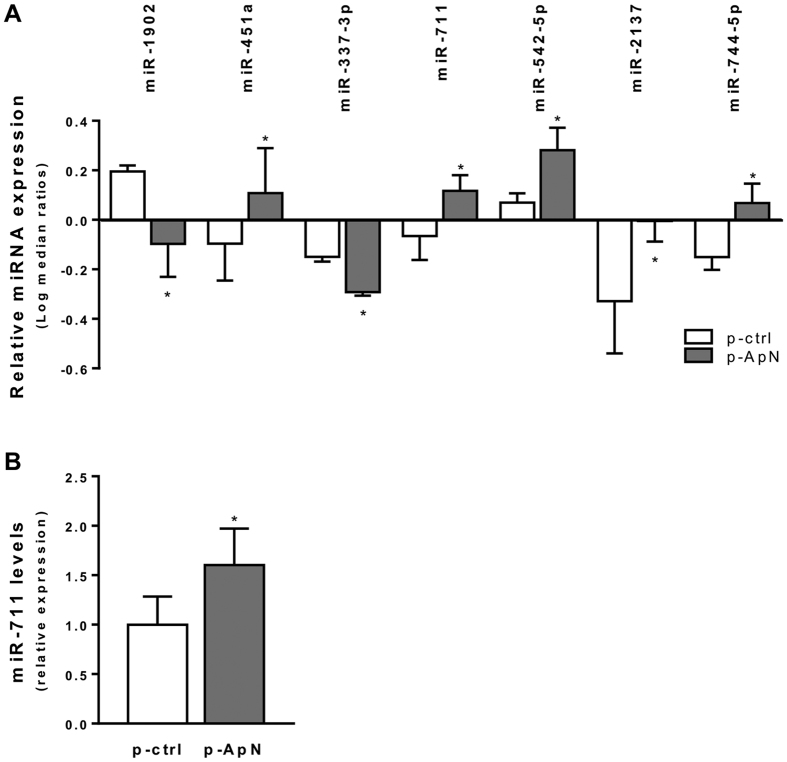
Effects of ApN gene electrotransfer on miRNA expression profiling in muscles of ApN-KO mice. MiRNA expression profiling (**A**) and quantification (**B**) were performed in muscles of ApN-KO mice transfected with the ApN gene, then challenged by LPS, as described in Fig. 1. MiRNA expression was screened by using microarrays, which contain 1157 mouse-specific miRNAs (**A**). Seven miRNAs showed different expression between the leg transfected with the ApN gene and the contralateral one receiving the control plasmid. Values are log-median ratio intensities for each sample relative to the common reference. Positive values indicate higher expression in the experimental sample than in the common reference and vice versa for negative values. Results are means ± SEM for 4 mice. *P < 0.05 for p-ApN *vs* p-ctrl (paired *t* test before applying the Benjamini-Hochberg procedure). These 7 miRNAs were next quantified by RT-qPCR (**B**). Out of these, only miR-711 was found to be significantly modified by ApN electrotransfer. Values were normalized to cyclophilin and presented as relative expression compared to p-ctrl levels. Results are means ± SEM for 9 mice. *P < 0.05 for the effect of the ApN gene.

Tibialis muscles of these mice were profiled by miRNA arrays (Fig. 2A). Among the 1209 miRNAs tested, the expression of 476 miRNAs was detectable in *tibialis*, whereas the expression of 7 miRNAs was differently regulated between the leg receiving the ApN gene and the contralateral one (P < 0.05 by paired *t*-test before applying the Benjamini-Hochberg procedure; data obtained in a limited number of mice, n = 4).

These 7 miRNAs were checked and quantified by real-time quantitative polymerase chain reaction (RT-qPCR) in a larger number of mice (n = 9). Only the expression of miR-711 was significantly modified by ApN gene electrotransfer and showed a ~50% upregulation, when compared to the empty plasmid electrotransfer (Fig. 2B). None of the miRNAs previously found to be regulated by ApN in adipose tissue^14^ were detected in muscle (data not shown), underlining the tissue-specificity of miRNA expression.

### MiR-711 expression in mice overexpressing ApN and in murine myotubes treated by the hormone

**Figure 3:**
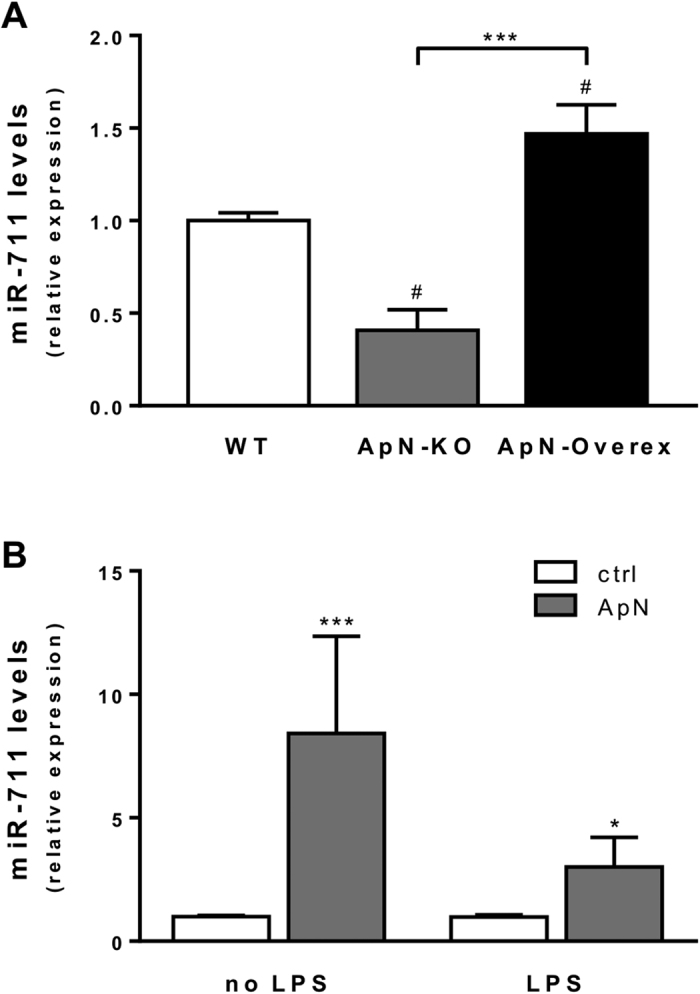
Upregulation of miR-711 expression in muscles of ApN-overexpressing mice (**A**) and in C2C12 myotubes after ApN treatment (**B**). (**A**) Quantification of miR-711 expression in *tibialis anterior* muscles from 3 groups of mice raised on the same genetic background: transgenic mice overexpressing ApN (ApN-Overex), their wild-type littermates (WT) and ApN-KO mice. All these mice were used in the basal state. Expression of miR-711 was quantified by RT-qPCR. Values were normalized to cyclophilin and presented as relative expression compared to WT values. Results are means ± SEM for 5–7 mice per group. ^#^*P* < 0.05 for WT vs ApN-KO or ApN-Overex mice; ***P < 0.001 for ApN-KO vs ApN-Overex mice. (**B**) miR-711 expression in differentiated C2C12 myotubes treated or not with 5 μg/ml ApN for 24 h and challenged or not with 1 μg/ml LPS for the last 20 h. Expression of miR-711 was quantified as described in (**A**) and presented as relative expression compared to basal conditions (i.e., no LPS and no ApN). Values are means ± SEM for 6 repeated experiments. *P < 0.05; ***P < 0.001 for ApN *vs* control. No significant effects for LPS *vs* no LPS.

To further strengthen our data obtained in ApN-KO mice after ApN electrotransfer, miRNA levels were also measured in transgenic mice overexpressing ApN (ApN-Overex) and in their wild-type (WT) littermates (controls). Because these mice were maintained on the same genetic background as ApN-KO mice, were of the same age, and sex and received the same diet, we compared the 3 groups of animals together. These mice were studied in basal conditions (i.e. without LPS). When compared to WT controls, transgenic mice overexpressing adiponectin (ApN-Overex mice) displayed a ~47% increase of miR-711 expression, while ApN-KO mice presented a reverse pattern with a ~60% decrease of expression (Fig. 3A).

MiR-711 mRNA levels were also measured *in vitro* in C2C12 myotubes. We studied the direct effect of ApN treatment on these cells, which were or not challenged by LPS. ApN treatment also upregulated miR-711 in these cells by ~3- (with LPS) to ~8-fold (without LPS) (Fig. 3B). The expression of miRNA-711 seems therefore to be dependent on the presence of ApN.

### In silico functional analysis of potential miR-711 target genes

In order to get some insights into the potential role of miR-711, we identified its predicted target genes using TargetScan algorithm. This algorithm generated 1216 potential targets genes.

We further annotated the biological function of these target genes using the GENECODIS database. Although several genes (n = 34) were involved in cancer pathways, we rather focused on target genes implicated in inflammation/immune responses. Nine genes were enriched for the Toll-like receptor (TLR) signaling pathway (KEGG, *P* < 0.01). Twelve other predicted target genes were also enriched for the vascular endothelial growth factor (VEGF) signaling pathway (KEGG, *P* < 0. 001), 13 for the T cell receptor pathway (KEGG, *P* < 0.001) and 11 for the chemokine signaling pathway (KEGG, *P* < 0.05).

Because in most of our experimental conditions, mice or cells were challenged by LPS, we chose to investigate the target genes belonging to the TLR4 signaling pathway. Among the 9 genes enriched for the TLR, 8 were predicted by a computational analysis as being involved into the TLR4 pathway and were further studied: TOLLIP (Toll interacting protein), FADD (Fas-associated protein with death domain), TAB1 (Transforming growth factor beta (TGF-β) activated kinase 1-Binding Protein 1), MAPK10 (Mitogen-activated protein kinase 10), PI3Kγ (Phosphoinositide-3-Kinase, Regulatory Subunit 3 Gamma), PI3Kδ (Phosphatidylinositol-4,5-Bisphosphate 3-Kinase, Catalytic Subunit Delta), the serine-threonine protein kinase encoded by the AKT1 gene and TNFα. The Supplementary Fig. 1. shows predicted base complementarities of miR-711 to the TLR4 target genes: only genes, which were next validated *in vitro* (see below) are represented.

### Involvement of target genes of miR-711 in the TLR4 pathway *in vitro*

Target genes of miR-711, belonging to the TLR4 pathway, were then further validated *in vitro*. To this end, we used a gain- or loss-of function approach in LPS-inflamed C2C12 myotubes transfected with miR-711 mimic or inhibitor (anti-miR), while being treated or not by ApN.

**Figure 4:**
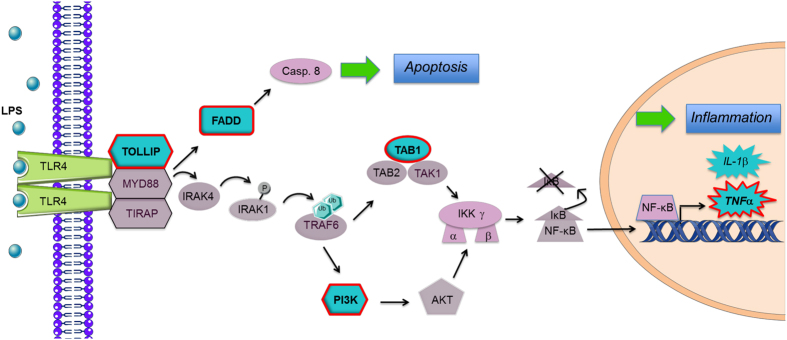
Localisation of target genes of miR-711 in the TLR4 signaling pathway. The target genes are surrounded in red. LPS activation of TLR4-mediated MyD88 (myeloid differentiation primary response 88)-dependent signaling pathway triggers the recruitment of its intracellular scaffold, which is comprised of three components MyD88, TIRAP (toll-interleukin 1 receptor domain-containing adaptor protein) and TOLLIP. IRAK4 (interleukin 1 receptor associated kinase 4) is then recruited to the complex, where it phosphorylates IRAK1, leading to its autophosphorylation and the subsequent ubiquitination of TRAF6 (TNF receptor associated factor 6). Ubiquitination of TRAF6 triggers its oligomerization and the recruitment and activation of the kinase TAK1 (TGF-beta activated kinase 1), in a complex with TAB1/2. These kinase complexes leads to the activation of the IKK (I-kappaB kinase) complex. The IKK complex phosphorylates IκB, resulting in IκB degradation, and permitting the release and the translocation of NF-κB into the nucleus. PI3K, through association with MyD88 or TRAF6, is also involved in NF-κB activation through an Akt-dependent mechanism leading to the phosphorylation and activation of the IKK complex. Together these events induce the expression of various pro-inflammatory genes such as TNFα or IL-1β through binding to κB sites. The MyD88 complex can also bind to FADD leading to the recruitment and the activation of caspase 8, which is known to induce apoptotic signals.

**Figure 5:**
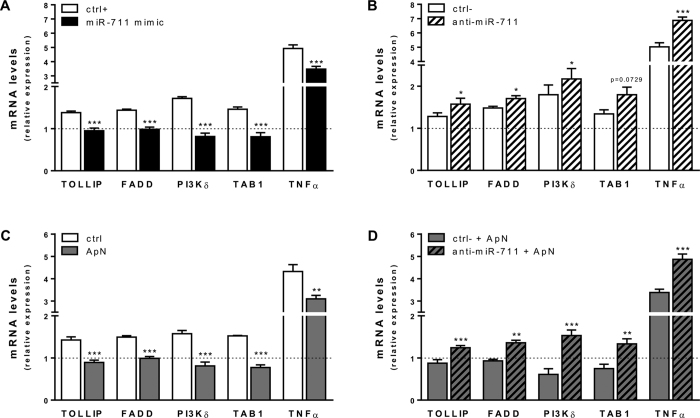
Involvement of target genes of miR-711 in the TLR4 pathway *in vitro*. C2C12 myotubes were either transfected with miR-711 mimic or its control (ctrl+) (**A**) or treated with ApN (**C**) for 24 h. Cells were also transfected with anti-miR-711 or its relative control (ctrl−) for 28 h (**B**), while ApN was added or not during the last 24 h (**D**). All conditions presented herein were obtained in C2C12 challenged by LPS except for the basal condition (no LPS, no transfection or any other treatments) represented by the dotted line. Only the 5 genes that turned out to be regulated by miR-711 are shown. MiRNA levels of TOLLIP, FADD, PI3Kδ, TAB1 and TNFα were quantified by RT-qPCR. Data were normalized to cyclophilin and presented as the relative expression compared to basal condition. Values are means ± SEM for 7 repeated experiments. *P < 0.05; **P < 0.01; ***P < 0.001 *vs* respective control.

Only 5 out of the 8 predicted target genes turned out to be targets of miR-711 and are illustrated in Fig. 4: TOLLIP, FADD, TAB1, PI3Kδ and TNFα. Expression of these target genes was increased in C2C12 cells inflamed by LPS (compare white columns to the horizontal dotted line corresponding to the basal situation without any treatments). Overexpression of miR-711 downregulated the expression of these 5 genes (Fig. 5A). Moreover, miR-711 mimic showed anti-inflammatory effects similar to those of ApN. As expected, blockade of miR-711 induced an opposite effect to the mimic and up-regulated the expression of all the 5 genes (Fig. 5B). Furthermore, ApN pretreatment attenuated LPS-induced stimulation of all these mRNAs, whereas anti-miR-711 reversed this preventive effect (Fig. 5C,D).

### MiR-711 as a mediator of the anti-inflammatory action of ApN on downstream pro-inflammatory molecules

**Figure 6:**
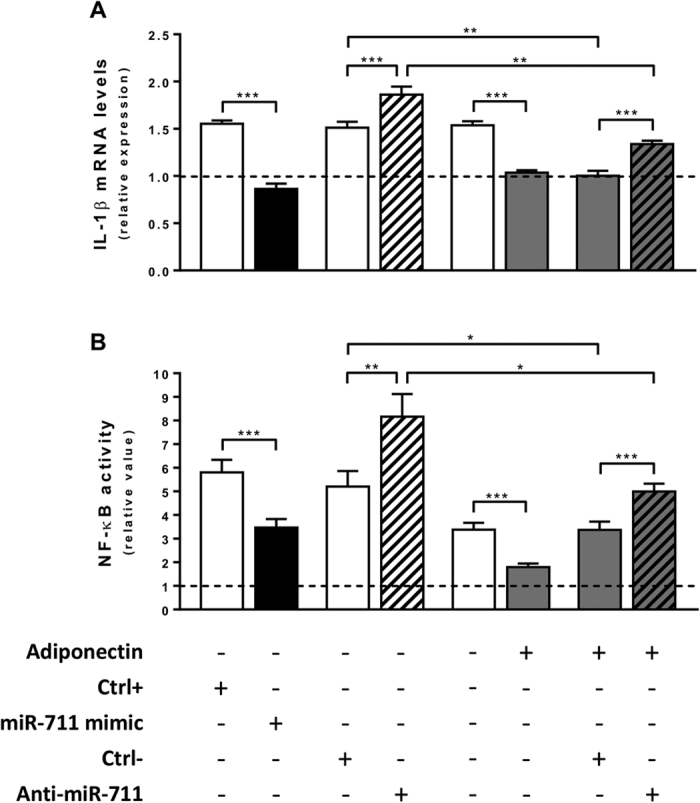
MiR-711 as a mediator of the anti-inflammatory action of ApN on downstream pro-inflammatory molecules *in vitro*. Cells were challenged by LPS and transfected with miR-711 mimic or anti-miR-711 and their respective controls (ctrl+ and−) (first 2 pairs of columns) and treated or not with ApN (last two pairs of columns). (**A**), Expression of IL-1β was quantified by RT-qPCR. Values were normalized to cyclophilin and expressed relative to basal condition (no LPS, no transfection or any other treatments) represented by the dotted line. (**B**), NF-κB luciferase reporter plasmid was transfected into C2C12 myotubes during 24 h. The medium was next renewed and cells were treated as described above. Luciferase activity was quantified and presented as relative values compared to the basal condition (the same as described above, except that NF-κB luciferase reporter plasmid had already been transfected). Values are means ± SEM for 7 repeated experiments. *P < 0.05; **P < 0.01; ***P < 0.001 for indicated conditions.

We took advantage of the experimental approach described above to study IL-1β, another pro-inflammatory cytokine that behaved like TNFα and was similarly downregulated by ApN electrotransfer *in vivo* (see Fig. 1). In inflamed C2C12 myotubes, overexpression of miR-711 downregulated IL-1β mRNA levels, while miR-711 silencing upregulated these levels. Moreover, the anti-inflammatory effects of ApN were reversed by the anti-miR (Fig. 6A).

We also studied the activity of NF-κB, a pleiotropic transcription factor, which plays a crucial role in inflammation and has been shown to be downregulated by ApN^8^. To this end, a NF-κB luciferase reporter plasmid was transfected into C2C12 myotubes 24 h before the treatments mentioned above. In these conditions, NF-κB activity displayed responses to miR-711 and to ApN roughly similar to those of IL-1β and the predicted target genes. Thus, both ApN and miR-711 mimic inhibited NF-κB activity, whereas blockade of miR-711 induced an opposite effect and further abolished the beneficial effect of ApN (Fig. 6B).

### MiR-711 as an anti-inflammatory agent of muscle inflammation *in vivo*

We next studied whether miR-711 was also effective *in vivo*. To this end, one *tibialis anterior* muscle was injected with a pre-miR-711 plasmid (p-miR-711) and the contralateral muscle with an empty control plasmid (p-ctrl); muscles were then electroporated. Nine days later, mice were challenged by LPS.

**Figure 7:**
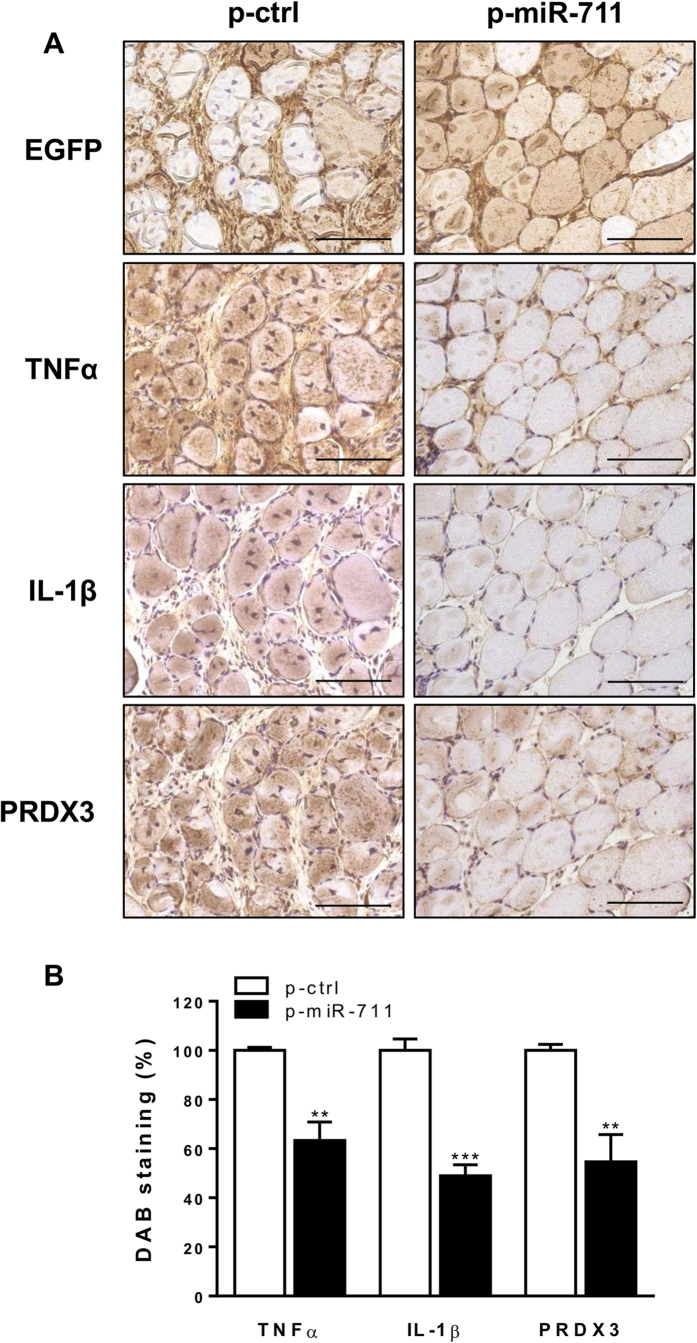
*In vivo* effects of pre-miR-711 electrotransfer on different markers of inflammation and oxidative stress in muscles of ApN-KO mice. One *tibialis anterior* muscle of ApN-KO mice was injected and electroporated with pre-miR-711-containing plasmid (p-miR-711), whereas the contralateral muscle was injected and electroporated with a control plasmid (p-ctrl). Both plasmids also contained the EGFP reporter gene. Nine days later, mice were challenged by LPS and the *tibialis anterior* muscles were sampled 24 h later. (**A**) Immunochemistry performed with specific antibodies directed against EGFP, TNFα, IL-1β and PRDX3. Scale bar = 100 μm. (**B**) Quantification of DAB staining areas within muscles. Results are means ± SEM for 5 mice (the contralateral muscle being used as control). **P < 0.01, ***P < 0.001 for the effect of miR-711.

Both legs were efficiently injected by plasmids as shown by Enhanced Green Fluorescent Protein (EGFP) immunostaining. When compared to the control one, the *tibialis anterior* muscle electroporated with pre-miR-711 displayed a ~40 to 50% decrease in DAB staining for TNFα, IL-1β and PRDX3, thereby indicating its anti-inflammatory action (Fig. 7A,B).

### Adiponectin enhances miR-711 expression in human myotubes *in vitro*

Finally, we tested the direct effect of ApN in primary cultures of human myotubes.

**Figure 8:**
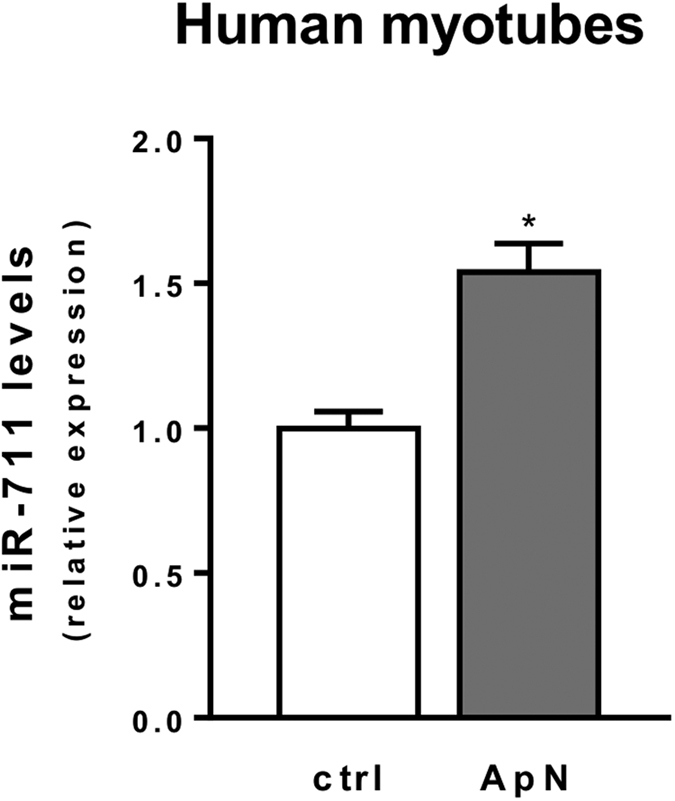
Effects of adiponectin on miR-711 expression on human myotubes. Primary cultures of human and healthy skeletal muscle cells were initiated from satellite cells obtained from Myobank-AFM. Differentiated myotubes were treated or not with 5 μg/ml ApN for 24 h. Expression of human miR-711 was quantified by RT-qPCR. Values were normalized to TBP and presented as relative expression compared to control condition (i.e. no ApN). Values are means ± SEM for 5 repeated cultures derived from 3 subjects. *P < 0.05 for the effect of ApN.

As in mouse, ApN is able to upregulate by 50% the expression of human miR-711 (hsa-miR-711) (Fig. 8). MiR-711 is a highly conserved microRNA^17^, which has only two different nucleotides between the human and murine forms. This suggests that hsa-miR-711 may be also involved in the anti-inflammatory effects of ApN in human skeletal muscle.

Except for TNFα, the predicted target genes of hsa-miR-711, identified by HumanTargetScan algorithm, were similar to those described in mice. We thus found that the 4 genes regulated by miR-711 in mouse were also on the list of potential target genes for human TLR4 signaling. This result opens new perspectives to treat skeletal muscle inflammation.

## Discussion

Muscle inflammation is a common worsening event occurring in metabolic disorders as well as in myopathies. Since ApN has anti-inflammatory effects on muscle and since its circulating levels are decreased in the metabolic syndrome^5^ as well as in muscle dystrophy^8^,^18^, we decided to explore whether miRNAs regulated by ApN may represent novel mechanisms for controlling muscle inflammation. By screening arrays, we found miR-711 as a strong candidate for mediating the anti-inflammatory action of ApN. Thus, ApN-KO mice showed decreased muscular expression of miR-711 together with enhanced inflammation and oxidative stress markers, while mice overexpressing ApN showed increased miR-711 levels (Fig. 3). Electrotransfer of the ApN gene in muscle of ApN-KO mice upregulated miR-711 while reducing inflammation and oxidative stress. Similar data were obtained in murine or human myotubes treated with ApN, indicating that ApN effects were direct and independent of any potential metabolic alteration induced by ApN deficiency or supplementation. Eventually, miR-711 overexpression recapitulated the anti-inflammatory effects of ApN both *in vitro* and *in vivo*, while miR-711 blockade had opposite effects. Taken together, our data indicate that miR-711 mediates ApN action on muscle.

We have previously shown that ApN exerts its beneficial effects on muscle via a signaling pathway involving AMPK (5′ adenosine monophosphate-activated protein kinase), a deacetylase Sirtuin (SIRT) 1, and the peroxisome proliferator-activated receptor-γ (PPARγ) coactivator-1α (PGC-1α)^8^. The exact mechanisms by which ApN upregulates miR-711 are still unsettled. However, according to UCSC Genome database (see methods), the miR-711 precursor is located across exon-intron junction of the collagen type VII alpha 1 (COL7A1) gene and is therefore likely to use the transcriptional machinery of the host gene. Moreover, the activator protein 1 complex (AP-1), which is an essential transcription factor of the COL7A1 gene^19^, is also a partner of PGC-1α in regulating gene program in muscle^20^. This could link ApN to PGC-1α/AP-1 and ultimately to miR-711.

So far, there are only very few reports about the role of miR-711. Yet, its modulation yielded beneficial effects on the heart, which are consonant with our data on skeletal muscle. Thus, activation of PPARγ, which is known to induce ApN production in both myocardial and skeletal muscle^21^,^22^,^23^, upregulated miR-711 in rats with myocardial infarction, thereby reducing cardiac fibrosis^24^. Moreover, cardiac ischemic preconditioning in mice, which provides cardioprotection against a subsequent ischemia/reperfusion injury, resulted in modulation of miR-711 levels, in a NF-кB-dependent manner^25^.

In order to get more insight into the role of miR-711, we identified its predicted target genes and focused on those belonging to the TLR4 signaling pathway as mice were challenged by LPS and the innate immune system plays a crucial pathogenic role in inflammatory muscle disease. Thus, increased TLR4-driven signalling in muscle from insulin-resistant obese and/or type 2 diabetic subjects contributes to worsening insulin-resistance and inflammation^26^. On the other hand, TLR4 ablation in myopathic mice slowed disease progression^27^. Five target genes of miR-711, which were predicted by computational analysis were then validated *in vitro*: TOLLIP, FADD, PI3Kδ, TAB1, and TNFα (Fig. 4). Upon activation, TLRs recruit adaptor proteins that give rise to specific TLR-mediated downstream signaling^28^. The Toll-interacting protein, is an adaptor that modulates TLR signaling. On one hand, overexpression of TOLLIP inhibits TLR-mediated NF-κB activation^29^, while on the other hand TOLLIP deficiency results in reduced production of pro-inflammatory cytokines in response to LPS^30^. In line with the latter observation, TOLLIP promotes inflammatory and apoptotic responses after myocardial infarction, leading to increased mortality^31^. At this time, the role of TOLLIP into skeletal muscle has not yet been studied. However, TOLLIP as other TLR4 signaling members appears to be strongly expressed in this tissue^32^. FADD is another adaptor molecule, which modulates apoptosis and necroptosis signaling through its associated effector caspase-8^33^. FADD can activate the NF-κB pathway^34^ and FADD-deficient mice showed impaired IL-1β production when challenged by LPS^33^. After delivering signals through adaptors, TLRs activate downstream kinases. PI3Kδ is one of four Class I PI3K isoforms. PI3Ks regulate several key events in inflammatory response to damage and infection^35^. It is now well-known that PI3K may activate Akt (protein kinase B), which mediates I-kappaB kinase alpha (IKKα) phosphorylation, thereby promoting NF-κB activation^36^. The protein kinase complex composed of TAK1 (TGFβ-activated kinase) and its associated binding proteins −1 and-2 (TAB1 and TAB2) also phosphorylates IKK, leading to NF-κB activation and inflammation^37^. TNFα is also one of the 5 target genes repressed by miR-711 mimic. This inhibition is of importance as TNFα plays a critical role in low grade-inflammation that accompanies muscle insulin-resistance and obesity^38^, and is also a potential target of therapy for inflammatory myopathies^39^.

Given the description of these target genes, we then hypothesized that miR-711 directly reduced the transcriptional activity of NF-κB, thereby repressing the expression of pro-inflammatory cytokines. We found that both ApN and miR-711 mimic actually inhibited NF-κB activity. IL-1β was reduced as a result from NF-κB inhibition. The attenuation of TNFα could be ascribed to either NF-κB inhibition or direct suppression by the miR-711, as seen above. Conversely, miR-711 blockade induced opposite effects and abolished the anti-inflammatory action of ApN *in vitro*.

*In vivo* experiments using muscle electrotransfer of pre-miR-711 recapitulated the anti-inflammatory effects observed *in vitro*. Thus, muscular electrotransfer of pre-miR-711 attenuated pro-inflammatory and oxidative stress markers, when compared to the contralateral leg transfected by a scrambled sequence. Moreover, this improvement was quite similar to that found after ApN electrotransfer.

In contrast to the clear-cut anti-inflammatory effects of ApN on most tissues, its role on muscle-adjacent tissues is still debated. Pro-inflammatory effects of ApN have been described *in vitro* in both human and murine synovial fibroblasts and chondrocytes^40^,^41^. However, systemic or local Apn strikingly mitigated the severity of the arthritis in the collagen-induced arthritis mouse model^42^,^43^. Thus, the effects of ApN on joints may be dependent on the *in vitro vs* the *in vivo* context, the environmental clues such as other cytokines or accompanying inflammation, as well as the exposure time (1 or 2 days *in vitro*^40^,^41^ *vs* up to 2 to 6 weeks *in vivo*^42^,^43^).

In skeletal muscle, ApN clearly upregulates miR-711, which in turn downregulates several components of the TLR4 pathway (and possibly of other pathways). This ultimately leads to repression of NF-κB activity and downstream pro-inflammatory cytokines, thereby relieving muscle inflammation. Our data may be extended to humans because of the high similarity of miR-711 between human and rodent species and because ApN also upregulated miR-711 levels in human primary myotubes.

In conclusion, we have identified miR-711 as a microRNA upregulated by ApN in skeletal muscle. MiR-711 served as a major mediator for ApN anti-inflammatory action by strongly repressing TLR4 signaling. This novel miRNA and its related target genes may open new therapeutic perspectives for controlling low-grade inflammation occurring in the metabolic syndrome as well as the more severe form prevailing in devastating myopathies. Future work is required to investigate the potential beneficial actions of miR-711 in dystrophic phenotype.

## Materials and Methods

### Animals

ApN-KO mice, which are characterized by a generalized lack of ApN, were obtained from Maeda *et al*.^9^ and maintained on a C57BL6/J background. In one experiment, transgenic C57BL/6 J mice overexpressing adiponectin (ApN-Overex mice) and their wild-type (WT) littermates were also used. ApN-Overex mice have been generated in our lab: native full-length ApN was placed under the control of the adipocyte aP2 promoter and because ApN is secreted, these mice showed a moderate elevation of circulating ApN levels^44^.

Mice were housed at a constant temperature (22 °C) with a fixed 12h-light, 12h-dark cycle. These animals received *ad libitum* a standard diet (Rat and Mouse n°3 Breeding, Specials Diets Services, Witham, UK) and were studied at the age of 12 weeks.

ApN-KO mice were submitted to muscle electrotransfer of the ApN gene. Animals were anaesthetized with a mixture of ketamine (75 mg/kg BW; Ketalar, Pfizer, New York, NY) and xylazine hydrochloride (10 mg/kg BW; Sigma-Aldrich, Bornem, Belgium) administered by ip injection. One *tibialis anterior* muscle was injected and electroporated with a plasmid coding for the ApN gene (p-ApN) or for the pre-miR-711 (p-miR-711), while the contralateral one received its respective control plasmid (p-ctrl). Nine days after electrotransfer, ApN-KO mice were challenged or not by LPS (4 mg/kg BW; serotype 0127:B8 from E.Coli; Sigma-Aldrich) and sacrificed 24 h later. Because LPS-injected mice reduced their daily food intake by 95% in our preliminary experiments, mice were fasted after the intraperitoneal injection (saline/LPS).

At the end of the experiments, mice were sacrificed by cervical dislocation (between 09.00 and 11.00 h). Blood samples were saved. Pairs of *tibialis anterior* muscles were weighed, frozen in liquid nitrogen and stored at −80 °C for subsequent analyses.

All experimental procedures were performed in accordance with the regulatory guidelines and regulations of the Ethical Committee for Animal Experimentation from the Medical Sector at the Catholic University of Louvain (n° LA1230396).

### Muscular electrotransfer of ApN gene or pre-miR-711 into muscle

#### Expression plasmids and DNA preparation

The plasmid encoding the ApN gene (p-ApN) was constructed by inserting the mouse full-length ApN cDNA^45^ into the pcDNA™3.1D/V5-His-TOPO^®^ vector (pcDNA™3.1 Directional TOPO^®^ Expression Kit from Invitrogen, Thermo Fisher Scientific, Gent, Belgium). An empty plasmid was used as control. The plasmid containing the precursor miRNA for *Mus musculus* miR-711 stem-loop with Enhanced Green Fluorescent Protein (EGFP) reporter gene (p-miR-711) as well as its negative control were purchased (GeneCopoeia, Rockville, USA). All plasmids were amplified in Escherichia coli top 10F’ (Invitrogen), purified with an EndoFree plasmid giga kit (Qiagen, Venlo, The Netherlands), and then were stocked at −80 °C.

### DNA injection and electroporation

30 μl of each plasmid solution (1.5 μg/μl) were injected into each *tibialis anterior*. Muscles were then electroporated by using transcutaneous electric pulses (8 square-wave pulses of 200 V/cm and 20 ms per pulse at 2 Hz) that were applied by two 8 mm spaced electrodes. Pulses were delivered by a Cliniporator system (Cliniporator, IGEA, Carpi, Italy), as described^10^,^11^,^46^.

### Immunohistochemistry and morphometry

Muscle samples were fixed in 10% formalin for 24 h and embedded in paraffin. 5 μm-thick sections were stained with hematoxylin. Sections were processed as previously described^8^,^10^ using rabbit polyclonal antibodies directed against PRDX3 (dilution 1:350, incubation 2h30) [gift from B. Knoops, University of Louvain, Brussels, Belgium^47^], TNFα (1:100, 2h30), IL-1β (1:300, 2h30) [both from Abcam, Cambridge, UK]. Rat monoclonal antibodies directed against ApN (1:5000, 2h30) or EGFP (1:1000, 2h30) were also used [both from Bio-connect, Huissen, Nederland]. Before immunostaining, sections were submitted to heat-mediated antigen retrieval using a microwave oven and Tris-citrate buffer (pH6.5). Binding of antibodies was detected by applying for 30 min at room temperature a second antibody, which was a biotinylated goat anti-rabbit IgG (H + L) or a biotinylated rabbit anti-rat IgG (H + L) (Labconsult, Brussels, Belgium). Peroxidase activity was revealed with DAB (Thermo Fisher Scientific), which produces a brown staining. For each marker, all slides from each *tibialis anterior* were treated simultaneously for immunohistochemistry analysis and DAB revelation, and then analyzed together. Immunohistochemical controls were performed by omission of the first antibody or of the first and second antibodies or by using pre-immune serum. For quantification, whole muscle sections were scanned using the Leica SCN400 slide scanner (Leica microsystems, Diegem, Belgium), and then the percentage of DAB surface area within muscle fibers was quantified using the Tissue Image Analysis 2.0 (Leica).

### MicroRNA array profiling

Total RNA of *tibialis anterior* muscle was extracted by the mirVana™ miRNA isolation kit (Thermo Fisher Scientific). 1 μg RNA from experimental samples and from a common reference mouse sample was labelled with Hy3™ and Hy5™ fluorescent probes, respectively (Exiqon, Vedbaek, Denmark). Each pair of labelled experimental and reference samples was mixed and hybridized to the miRCURY™ LNA Array (7th generation version; Exiqon), which contains 4 replicates of each of the 1157 mouse-specific miRNA probes (based on mirBASE version 19.0). The quantified signals were background corrected and normalized using the global Lowess (LOcally WEighted Scatterplot Smoothing) regression algorithm within each array, to adjust for any intensity-dependent dye bias.

### Cell culture and transfection of miR mimic or anti-miR *in vitro*

#### Culture of murine C2C12 cell line

C2C12 myoblasts were cultured as previously described^23^. Briefly, after proliferation, cells were cultured in basal medium (high glucose Dulbecco’s Modified Eagle Medium (DMEM) +2% heat-inactivated horse serum (HS)) for 5 days to induce myogenic differentiation. Myotubes were used at day 5. Myotubes were pre-treated or not with 5 μg/ml mouse full-length recombinant ApN (Biovendor GmbH, Heidelberg, Germany) added to the basal medium for 24 h, while being challenged by 1 μg/ml of ultrapure LPS from E.coli K12 (Invivogen, Toulouse, France) for the last 20 h. These conditions were chosen according to a previous work^14^ and to our own preliminary data. At the end of the culture, cells were washed in ice-cold phosphate-buffered saline (PBS) before RNA or nuclear extraction.

#### Transfection of miR-711 mimic or inhibitor in C2C12 cells

Synthetic double-stranded oligonucleotide mimicking mature endogenous miR-711 (miR-711 mimic, 5 nM), miR-mimic negative control (AllStars Negative Control, ctrl+, 5 nM), Anti-miR-711 single-stranded oligonucleotide (Anti-miR, 50 nM) or Anti-miR negative control (miScript Inhibitor Negative Control, ctrl-, 50 nM) (all from Qiagen) were delivered into mature myotubes (day 5). Delivery was performed by using Dharmafect 3 siRNA transfection reagent (Dharmacon, Lafayette, CO). Cells and media were collected 24 h (mimic) or 28 h (anti-miR) post transfection. In some experiments, 4 h after initiation of transfection with Anti-miR or its negative control, cells were treated with or without 5 μg/ml ApN for 24 h, while being or not challenged by 1 μg/ml LPS for the last 20 h.

#### Culture of human myotubes

Primary cultures of human skeletal muscle cells were initiated from satellite cells obtained from healthy subjects via Myobank-AFM (Association Française contre les Myopathies). Culture experiments were done in duplicate and the data from a given individual were then averaged.

Cultures were performed as described^23^ with minor modifications. Myoblasts were grown in 35-mm plates at 37 °C in the presence of 5% CO_2_ in F-12 (Ham) supplemented with 20% foetal bovine serum (FBS), 1% L-glutamine, and 100 μg/ml Primocin^TM^ (Invivogen) (all other products from Thermo Fisher Scientific). After proliferation, at the end of which the seeding density has reached 70–80%, the growth medium was replaced by the fusion medium which consists of 1 part DMEM, 1 part F-12 (Ham), 2% HS, 1% L-glutamine, and Primocin^TM^ (Life Technologies). This fusion medium was then changed every 2 days, and differentiation was allowed to continue for 11 days (time required to obtain mature myotubes with characteristic elongated and multinucleated morphology) before the experimentation period. Cells were next treated with or without human recombinant ApN (5 μg/ml) for 24 h (Biovendor). At the end of the experiments, cells were collected and rinsed twice in PBS before miRNA extraction. All experiments involving human cells were performed in accordance with approved guidelines and regulations of the bioethics department of the Direction Générale de la Recherche et de l’Innovation (DGRI; n° AC-2013–1868).

### Direct miRNA or mRNA quantification by RT-qPCR

MiRNA and RNA were isolated from cultured cells with TriPure reagent (Roche Diagnostics, Vilvoorde, Belgium).

For miRNA quantification, 1 μg total RNA were reverse transcribed by using the NCode™ VILO™ miRNA cDNA Synthesis Kit (Thermo Fisher Scientific). 10 ng of total RNA equivalents were amplified with iQSyber Green Supermix (Bio-Rad Laboratories, UK Ltd., Hertfordshire, UK) using commercial miRNA-specific forward primers (QIAGEN, Venlo, The Netherlands) and a reverse universal primer (provided in the NCode VILO miRNA cDNA synthesis kit).

For mRNA quantification, 2 μg of total RNA were reverse transcribed as described previously^48^, performed with designed primers (Supplemental Table 1).

The threshold cycles (Ct) were measured in separate tubes and in duplicate. To ensure the quality of the measurements, each plate included a negative control for each set of primers and analysis of the melting curve was carried out at the end of the amplification. Cyclophilin (mouse) and TATA-box binding protein (TBP, human) were used as reporter genes. Relative changes in the expression level of one specific gene were presented as 2^−ΔΔCt ^48.

### NF-κB activity measured by a luciferase reporter assay

PathDetect NF-κB *cis*-Reporting System (Stratagene; Agilent Technologies, Santa Clara, USA); including pNF-kB-Luc *Cis*-Reporter Plasmid which contains the NF-κB binding element fused to the firefly luciferase gene, and pFC-MEKK Control Plasmid which contains the SV40 promoter fused to the firefly luciferase gene; were used to measure the NF-κB activity. 1 μg of pNF-kB-Luc or pFC-MEKK was transfected into C2C12 myotubes for 24 h using Lipofectamine (Invitrogen). The medium was next renewed and treatments described above were performed. Luciferase activity was quantified by a luminometer 48 h after transfection using a Luciferase Assay Kit from Stratagene, according to the manufacturer’s instructions.

### In silico functional profiling of miR-711 target genes

Potential miR-711 target genes were predicted using TargetScan algorithms (version 6.2, http://www.targetscan.org/)^49^. Next, the biological function of target genes was annotated using GENECODIS database (version 3) by integrating Kyoto Encyclopedia of Genes and Genomes (KEGG) pathways and Gene Ontology (GO) Biological Process (http://genecodis.cnb.csic.es/)^50^.

### Genomic location of miR-711

MiR-711 location was investigated by UCSC (University of California Santa Cruz) Genome Browser (http://www.genome.ucsc.edu/).

### Presentation of the results and statistical analysis

The results are means ± SEM for the indicated numbers of mice or independent cultures. Comparisons of 3 groups of mice (WT, ApN-Overex and ApN-KO) were performed by one-way ANOVA followed by Tukey’s test (Prism 6; GraphPad Software, California, USA). Comparisons between electroporated muscle groups or some *in vitro* experiments were carried out using two-tailed paired Student’s *t*-test. The influence of ApN and that of LPS on miR-711 expression were assessed by two-way ANOVA with F test, followed by *post-hoc* two by two comparisons with Bonferroni correction for multiple comparisons (Prism 6). Differences were considered statistically significant at P < 0.05. Due to the high number of microRNAs being tested in parallel, the Benjamini and Hochberg multiple testing adjustment method has been applied by Exiqon to control the false discovery rate of the p-values (two-tailed paired Student’s *t*-test) of the microRNA array and no significantly differences were finally observed. Results presented Fig. 2 are p-values before applying the Benjamini-Hochberg procedure.

## Additional Information

**How to cite this article:** Boursereau, R. *et al*. New targets to alleviate skeletal muscle inflammation: role of microRNAs regulated by adiponectin. *Sci. Rep.* **7**, 43437; doi: 10.1038/srep43437 (2017).

**Publisher's note:** Springer Nature remains neutral with regard to jurisdictional claims in published maps and institutional affiliations.

## Supplementary Material

Supplementary Information
