# Supplementary material for: New targets to alleviate skeletal muscle inflammation: role of microRNAs regulated by adiponectin
Source: Sci Rep. 2017 Feb 27;7:43437. doi: 10.1038/srep43437 (PMC5327483; doi:10.1038/srep43437)
Supplement: Supplementary Information [file srep43437-s1.pdf]

New targets to control skeletal muscle inflammation: microRNAs regulated by adiponectin

Raphaël Boursereau, Michel Abou-Samra, Sophie Lecompte, Laurence Noel

and Sonia M. Brichard

Supplementary Fig. 1. Predicted base complementarities of murine miR-711 to the TLR4 target genes. The base complementarities of mature miR-711 to 3'UTR binding sites of TOLLIP, FADD, PI3Kδ, TAB1 and TNFα are predicted by TargetScan algorithms (version 6.2, http://www.targetscan.org/).

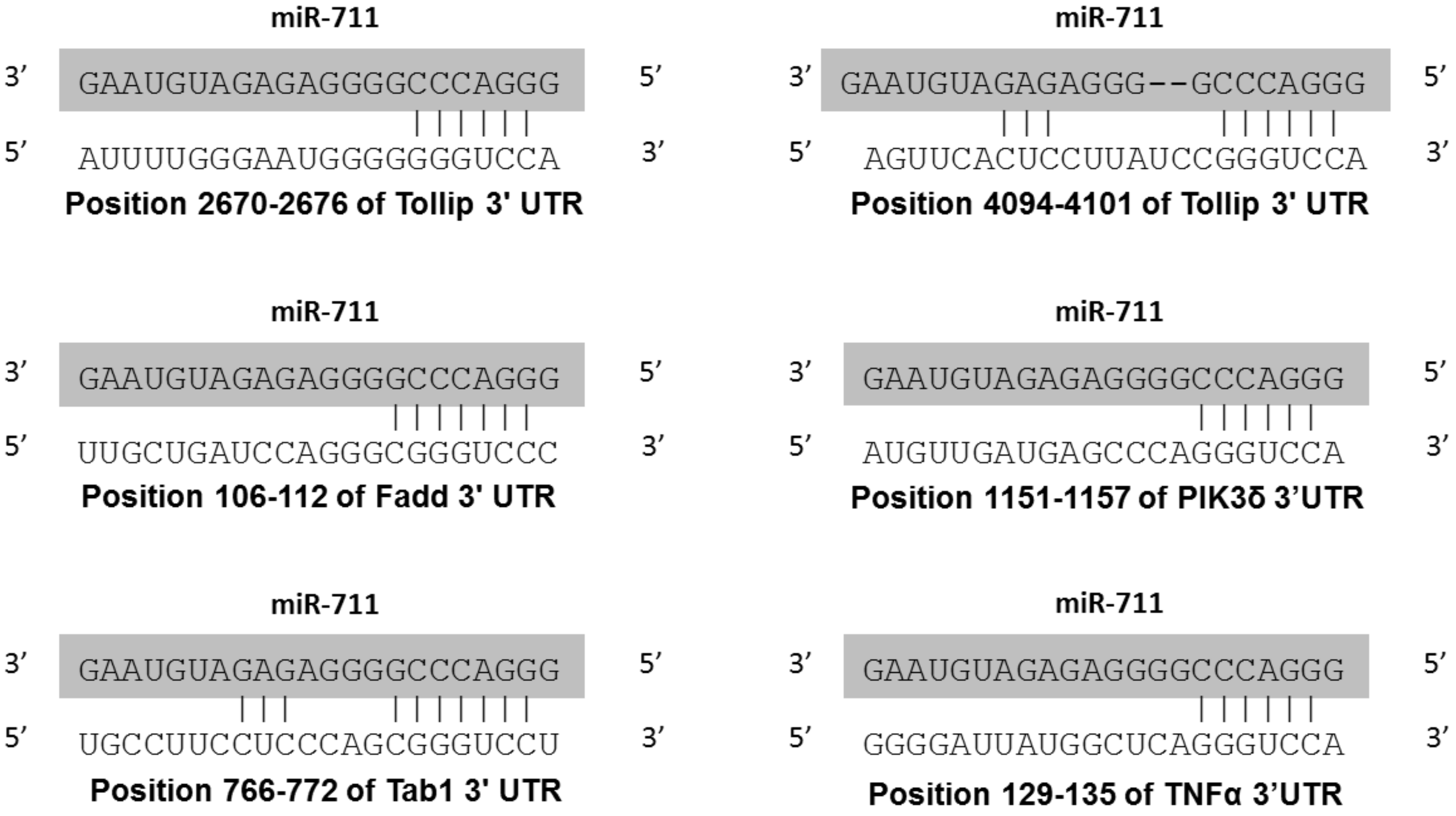

Supplementary Table 1: Gene sequences used as forward and reverse primers for RT-qPCR

Gene	Forward Primer	Reverse Primer
AKT1	GAGTCCTACCCCTGAATCTGCC	CCCAGGGATGATGCTCAGAGTC
FADD	CGAAGTCTGAGTGAGCGGGTAA	AGATTCCTGGGCTTCTTCCACC
IL-1β	GCTCTCCACCTCAATGGACAGA	GCCCAAGGCCACAGGTATTTTG
MAPK10	CGTAATCTTGTCACAGAAATCCCATAC	CCTGCTTCTCAGAAACACCCTTC
PI3Kγ	TGGGAAACCAGATGGTGCATTC	CGTGCTGTAGATGACACAGTGC
PI3Kδ	CGTGGTTGTTGACTTCTTGC	TGCCAATGAGGAGGCTGATCTG
TAB1	ACGCAGGGAAGATCAAGCAGAT	CGATGATGGGTTTGGACTTGGC
TOLLIP	GTATAGCCTGAGTGGGAGGCAG	AACACCCTGCTGGTACACAGTT
TNFα	GCCACCACGCTCTTCTGTCT	GTCTGGGCCATGGAACTGAT
Cyclophilin	AACCCCACCGTGTTCTTC	TGCCTTCTTTCACCTTCCC
TBP(H)	TTGGAGGGCAAGTCTGGTG	CCGCTCCCAAGATCCAACTA

All sequences are from murine species expect for TBP which is from Human (H).
